# Brain metastasis from non-small cell lung cancer: crosstalk between cancer cells and tumor microenvironment components

**DOI:** 10.1038/s12276-025-01604-z

**Published:** 2025-12-22

**Authors:** Myung-Seo Kim, Juyoun Lee, Jeong Eun Lee, Jong Hun An, Jun Young Heo, Min-Kyung Yeo

**Affiliations:** 1 Department of Pathology, Translational Immunology Institute, Chungnam National University School of Medicine, Daejeon, Republic of Korea; 2 System Network Inflammation Control Research Center, Chungnam National University, Daejeon, Republic of Korea; 3 Department of Neurology, Chungnam National University School of Medicine, Daejeon, Republic of Korea; 4 Department of Internal Medicine, Chungnam National University School of Medicine, Daejeon, Republic of Korea; 5 Department of Medical Science, Chungnam National University School of Medicine, Daejeon, Republic of Korea; 6 Department of Biochemistry, Chungnam National University School of Medicine, Daejeon, Republic of Korea

**Keywords:** Non-small-cell lung cancer, Metastasis

## Abstract

The majority of patients with lung cancer are diagnosed at an advanced stage, with a substantial proportion exhibiting signs of brain metastases (BMs). BM is associated with debilitating symptoms, including headaches, seizures and neurological or cognitive impairments, which severely impact the quality of life of patients. Despite considerable advancements in lung cancer treatment modalities, the management of BM remains challenging due to the complex cellular and structural nature of the blood–brain barrier and resistance driven by acquired genetic mutations. Non-small cell lung cancer (NSCLC) is characterized by diverse genetic alterations. The application of immunotherapy has successfully enhanced antitumor immune responses within the tumor microenvironment (TME) of affected patients. The intricate interplay between NSCLC cells and the TME plays a critical role in the pathogenesis of BM. This review focuses on the brain-specific TME and its exploitation by tumor cells to establish metastases through strategic, site-specific mechanisms. The reciprocal molecular interactions, immune modulation and adaptation of NSCLC cells to the brain metastatic niche are central to this process. A deeper understanding of the complex crosstalk between tumor cells and TME is essential for devising more effective and targeted therapeutic interventions for BM.

## Lung cancer metastasis exploits brain tumor microenvironment

Lung cancer is a major health issue, which complicates treatment and worsens patient outcomes. This article explores challenges of treating brain metastases in non-small cell lung cancer (NSCLC) and the role of the tumor microenvironment in this process. Researchers found that NSCLC cells use specific molecular pathways to invade the brain, overcoming barriers such as the blood–brain barrier. They studied how cancer cells interact with surrounding cells, to establish a supportive environment for tumor growth. The study highlights the importance of understanding these interactions to develop better treatments. The authors conclude that targeting the tumor microenvironment could lead to more effective therapies for patients with NSCLC with brain metastases. Future research may focus on new drug delivery methods across the blood–brain barrier and identifying blood-based biomarkers for early detection and monitoring of brain metastases in patients with lung cancer.

This summary was initially drafted using artificial intelligence, then revised and fact-checked by the author.

## Introduction

### Clinical relevance and unmet needs in BM of NSCLC

Lung cancer is the second most prevalent cancer type in developed countries, posing a substantial challenge owing to its high mortality and frequent recurrence rates [1]. The majority of patients (75%) are diagnosed at advanced stages (stage III or IV), often presenting with distant metastases, which limits the feasibility of curative treatments. Among the various primary tumors, lung cancer is the leading cause of brain metastasis (BM), constituting approximately 40–50% of all BM cases [2]. Given that BM is not only associated with high mortality but also neurological complications, such as seizures, the overall quality of life of patients is substantially impaired [3].

Despite ongoing advancements in lung cancer treatment options, including targeted therapies and chemoradiotherapy, the management of BM in non-small cell lung cancer (NSCLC) patients remains an important challenge. The blood–brain barrier (BBB) and complex cellular and structural factors limit drug delivery, often resulting in resistance driven by acquired mutations particularly within the brain microenvironment. The precise mechanisms underlying the metastatic cascade from lung to brain are still not fully understood. Therefore, it is crucial to investigate the mechanisms uniquely associated with BM in NSCLC.

### Distinct molecular mechanisms underlying BMs in lung cancer, breast cancer and melanoma

The majority of BM originate from lung cancer (40–50%), breast cancer (15–25%) and melanoma (5–20%) [2]. While the overall process of extravasation of tumor cells, traversal across the BBB and subsequent colonization is generally conserved across solid tumors, the specific molecular mechanisms differ among cancer types (Table 1). They have their distinct key molecular driver genes that promote BM, including NSCLC; epidermal growth factor receptor (EGFR), anaplastic lymphoma kinase (ALK), c-ros oncogene 1 (ROS1) and Kirsten rat sarcoma viral oncogene homolog (KRAS) mutations; small cell lung cancer (SCLC); MYC amplification, tumor protein p53 (TP53) and retinoblastoma transcriptional corepressor 1 (RB1) loss; breast cancer; human epidermal growth factor receptor 2 (HER2) amplification; phosphatidylinositol-4,5-bisphosphate 3-kinase catalytic subunit alpha (PIK3CA) mutation; BRCA1/2 alteration; melanoma; and B-Raf proto-oncogene serine/threonine kinase V600E (BRAF V600E), neuroblastoma RAS viral oncogene homolog (NRAS) and KIT proto-oncogene receptor tyrosine kinase mutation [4].

**Table 1:** Mechanisms of brain metastasis in NSCLC, breast cancer and melanoma.

	Lung cancer (NSCLC and SCLC)	Breast cancer	Melanoma
Incidence	~30–60% of cases	~15–20% of cases	~5–10% overall (high per-patient risk).
Growth patterns in brain	Often multiple, small parenchymal lesions (especially in SCLC); NSCLC metastases rely on vascular co-option and astrocytic support	Multiple parenchymal lesions, frequent leptomeningeal spread in HER2^+^; tumor–astrocyte gap junctions crucial for colonization	Hemorrhagic, angiogenic lesions; melanoma cells often co-opt pre-existing vasculature and show high propensity for bleeding
Leptomeningeal disease	Seen in a subset (notably EGFR/ALK).	Well-recognized in HER2 and other subtypes.	Occurs; carries poor prognosis.
Key molecular drivers	NSCLC: EGFR mutation, ALK rearrangement → strong CNS tropism; SCLC: neuroendocrine phenotype, early hematogenous spread	HER2-positive and triple-negative (basal-like) subtypes strongly predisposed to BM; HER2 overexpression enhances BBB crossing	BRAF V600 and NRAS mutations linked with high CNS tropism; MAPK pathway activation promotes BM
BBB interaction	Tumor-derived exosomes (EGFRvIII, miR-181c) disrupt tight junctions (claudin-5, occludin, ZO-1); VEGF/MMPs enhance permeability	COX2, HBEGF, ST6GALNAC5 promote endothelial adhesion and BBB transmigration; HER2^+^ cells induce vascular remodeling	Highly invasive phenotype; integrins (αvβ3), matrix metalloproteinases, and angiogenic factors disrupt BBB; melanoma cells often exploit hematogenous spread + high motility
Immune microenvironment	SCLC/NSCLC cells shape an immunosuppressive niche (astrocyte crosstalk, PD-L1 upregulation, T_reg_ and microglia recruitment)	Breast BM cells activate astrocytes via STAT3, forming tumor–astrocyte gap junctions that transfer cGAMP/IFN signals → prosurvival milieu	Melanoma BM has high immune infiltration but also immunosuppressive microglia/astrocyte signaling; strong capacity to exploit immune checkpoints (CTLA-4, PD-1 axis)
Adaptation for survival	Activation of PI3K/AKT, STAT3, WNT/β-catenin pathways; reliance on astrocytic support and immune evasion	HER2 signaling enhances survival; astrocytic STAT3/cytokines (IL-6, IFNα) protect tumor cells from apoptosis	MAPK pathway activity + metabolic plasticity; melanoma cells adapt to hypoxic brain niches and exploit neuronal-like signaling
Systemic therapies	EGFR Tyrosin kinase inhibitors (TKIs) (osimertinib; ± chemo FLAURA2) and ALK TKIs (alectinib, lorlatinib)	HER2CLIMB: tucatinib + trastuzumab + capecitabine improves intracranial outcomes and overall survival	Checkpoint blockade (nivolumab + ipilimumab; durable intracranial responses) and BRAF/MEK (dabrafenib + trametinib; high but less durable intracranial responses)
Radiation therapies	Stereotactic radiosurgery (SRS) preferred for limited lesions; Whole brain radiotherapy (WBRT) selectively.	Same; integrate with systemic HER2 therapy.	SRS commonly used; can be combined with immunotherapy or targeted therapy in trials. Follow histology-specific guidelines.
Prognosis	~7–47 months	~3–36 months	~5–34 months

NSCLC activate the phosphatidylinositol-3-kinase/protein kinase B (PI3K/AKT), signal transducer and activator of transcription 3 (STAT3) and WNT/β-catenin pathways, relying on astrocytic support and formation of an immune-suppressive metastatic niche6. Breast cancer cells enhance tumor survival by HER2 signaling, while astrocyte-derived STAT3 and cytokines such as interleukin-6 (IL-6) and interferon-alpha (IFNα) protect tumor cells from apoptosis. Melanoma progression is facilitated by strong mitogen-activated protein kinase (MAPK) pathway activity and has metabolic plasticity, enabling adaptation to hypoxic brain niches and exploiting neuron-like signaling for colonization and growth [4, 5].

NSCLC-derived exosomes and microRNAs (miRNAs) impair endothelial tight junctions (TJs), while vascular endothelial growth factor (VEGF) and matrix metalloproteinases (MMPs) further enhance permeability. In breast cancer, cyclooxygenase-2 (COX2), heparin-binding epidermal growth factor-like growth factor (HBEGF) and ST6 *N*-acetylgalactosaminide alpha-2,6-sialyltransferase 5 (ST6GALNAC5) facilitate endothelial adhesion and BBB transmigration. In melanoma, integrins, MMPs and angiogenic factors collectively disrupt BBB integrity [4, 5].

NSCLC establish an immunosuppressive niche through astrocyte crosstalk, programmed death-ligand 1 (PD-L1) upregulation, recruitment of regulatory T cells and microglia. In breast cancer, tumor cells activate astrocytes via signal transducer and activator of STAT3 signaling, leading to the formation of tumor–astrocyte gap junctions, similarly observed in SCLC. In melanoma, despite high levels of immune cell infiltration, tumor progression is sustained by immunosuppressive signaling from microglia and astrocytes, together with robust checkpoint pathway exploitation.

### Comparative mechanisms of BM between SCLC and NSCLC

Among lung cancers, SCLC and NSCLC exhibit distinct mechanisms underlying BM (Table 2). SCLC shows a high incidence of BMs (50–60%) at the time of diagnosis, largely owing to its propensity for early hematogenous spread. The high number of circulating tumor cells (CTCs) observed in SCLC reflects the small cell size and rapid proliferative rate. SCLC produces diffuse and multifocal BMs, frequently involving deep brain structures [6]. By contrast, NSCLC presents with a lower incidence of BMs at diagnosis; however, 40–50% of patients eventually develop BMs during the course of disease progression. NSCLC-related BMs are usually more localized and tend to occur in cortical regions.

**Table 2:** Comparative mechanisms of brain metastasis between SCLC and NSCLC.

	SCLC	NSCLC
Histology	Neuroendocrine features, small round morphology, high proliferation rate	Heterogeneous histology (adenocarcinoma, squamous, large cell), slower growth
Neuronal affinity	Neuronal adhesion molecules (NCAM, L1CAM) enhance brain tropism; neuron-specific enolase (NSE) supports invasion	Less neuronal mimicry; relies more on integrins, cadherins and angiogenic factors (VEGF, PlGF)
Dependence on neuronal activity	Glutamatergic signaling and neuronal electrical activity for growth	Depends more on angiogenesis and microenvironmental support
BBB interaction	SCLC cells exploit high plasticity and NGFR signaling to cross BBB	Disruption of BBB by VEGF/PlGF signaling, endothelial reprogramming, MFSD2A downregulation facilitates entry
Interaction with astrocytes	Strong crosstalk: reactive astrocytes promote tumor survival via STAT3 and neuroligin–neurexin signaling; tumor integrates into neuronal circuits	Astrocyte activation contributes to immunosuppression and metabolic support but less neuron-like integration
Immune microenvironment	Astrocyte-driven STAT signaling, microglia-mediated immune evasion	Crosstalk between cancer cells and TME cells play a crucial role in modulating the immunosuppression; atrocytes, immunosuppressive effects of TAM, immune modulation by T cells
Therapeutic implication	Poor response to ICIs in brain metastases; rapid relapse despite chemo/radiation; drug resistance common	EGFR/ALK TKIs with CNS penetration (osimertinib, lorlatinib, alectinib) effective; Immune checkpoint inhibitors (ICIs) show meaningful intracranial efficacy in PD-L1^+^ NSCLC
Clinical behavior	Early and aggressive brain metastasis; high frequency at diagnosis	Later occurrence than SCLC; dependent on oncogenic driver status

SCLC is a neuroendocrine carcinoma defined by small cell morphology, and its neuron-like properties facilitate BM. Several mechanisms contribute to this process: (1) neuronal adhesion molecules such as NCAM and L1CAM enhance tumor affinity for brain tissue, (2) neuron-specific enolase (NSE) promotes local invasion and (3) nerve growth factor receptor (NGFR)-dependent signaling drives tumor cell migration [6]. Once in the brain, SCLC cells closely interact with astrocytes and exploit neuronal activity as a mechanism of metastatic colonization. Reactive astrocytes foster tumor–neuron communication through the neuroligin–neurexin axis, enabling SCLC cells to integrate into neuronal circuits and hijack glutamatergic signaling and electrical activity. STAT signaling functions as a central mediator of tumor–astrocyte crosstalk, promoting immune suppression and accelerating metastatic progression in SCLC [6].

By contrast, NSCLC BMs are largely driven by oncogenic alterations such as EGFR, ALK and KRAS mutations, which promote tumor cell survival, proliferation and migration—and also serve as major therapeutic targets. NSCLC progression in the brain is shaped by complex tumor microenvironment (TME) interactions in which immune modulation and vascular endothelium play central roles. NSCLC-derived exosomes have been shown to remodel brain endothelial cells, thereby increasing BBB permeability and facilitating metastatic colonization.

Despite advances, the common molecular alterations (for example, TP53 and RB1 loss) of SCLC remain undruggable, rendering systemic chemo–immunotherapy often insufficient and whole-brain radiotherapy as the cornerstone of treatment [2]. By contrast, NSCLC harbors diverse genetic mutations with multiple actionable targets and exhibits notable immunosuppressive characteristics, making it a primary focus for the development of targeted and immunotherapy strategies. Favorable responses to immunotherapy have been reported in a subset of patients with NSCLC, thereby driving ongoing research efforts in this area.

These differences highlight the need for a deeper understanding of the TME in NSCLC as TME-driven mechanisms play a pivotal role in shaping metastatic behavior and therapeutic response. Comprehensive investigation of the TME is therefore essential for the development of innovative therapeutic strategies in NSCLC. In this review, we summarize current knowledge from the basic mechanisms to novel insight underlying BMs in NSCLC, with particular emphasis on the contribution of the tumor microenvironment.

### Current understanding of the pathogenesis of BM from lung cancer

The ‘seed-and-soil’ theory posits that metastatic cancer cells (the ‘seeds’) can survive and proliferate only when they encounter a congenial organ microenvironment (the ‘soil’), providing an explanation for the organ-specific nature of metastasis [7]. Disseminated tumor cells (DTCs) from lung cancer tend to favor colonization in suitable soil environments, such as brain, bone, liver and adrenal gland. Among these sites, NSCLC exhibits a high tendency to metastasize to the brain. In particular, the TME is believed to play an important role in influencing the dissemination, progression and metastasis of cancer to target organs.

The process of lung cancer progression to BM can be categorized into four main stages: (1) departure of tumor cells from primary lung cancer and preparation of the premetastatic niche (PMN): lung cancer cells invade surrounding tissues and exit the primary site through TME-induced epithelial–mesenchymal transition (EMT) [8]. Mesenchymal transition allows cancer cells to acquire motility and invasive properties, accompanied by altered expression of cell adhesion and cytoskeleton [8]. Before invasion, lung cancer cells can initiate the formation of a unique microenvironment in the brain, thereby establishing a metastasis-conducive environment termed PMN. The interplay between lung cancer cells and TME of the primary site contributes to the development of PMN via modulation of the systemic immune response [9]. (2) Intravasation and circulating tumor microemboli (CTM) formation: lung cancer cells enter the circulation and form hypoxic CTC clusters, known as CTM [10]. CTMs are capable of resistance to physical stress and apoptotic signals in the bloodstream through the protective influence of the TME surrounding cancer cells, eventually target the brain [11]. (3) BBB penetration: infiltration of the BBB is considered a rate-limiting step in the formation of BM. Lung cancer and TME cells secrete angiogenic and extracellular matrix (ECM) degradation factors that function to increase BBB vascular permeability [12]. (4) Colonization in the brain parenchyma: after breaching the BBB and entering the brain parenchyma, cancer cells interact with brain-resident cells, including astrocytes and microglia, contributing to the expansion of both tumor and TME cell populations [13]. This process involves complex reciprocal communication, marking the final stage of BM. This study highlights the metastasis-specific roles of the TME at each stage of the metastatic cascade, with the aim of providing deeper insights into the process of BM beyond the primary tumor.

While intrinsic tumor cell properties contribute to metastasis, the surrounding TME critically influences the development and progression of BM. As a metastatic site, the brain presents unique anatomical and physiological barriers, highlighting the essential need for further understanding of the brain-specific TME. The cells and components involved in this process are listed in Supplementary Fig. 1 and will be described in each corresponding figures.

## PMN

### Preparation of the metastatic environment: PMN formation

**Fig. 1:**
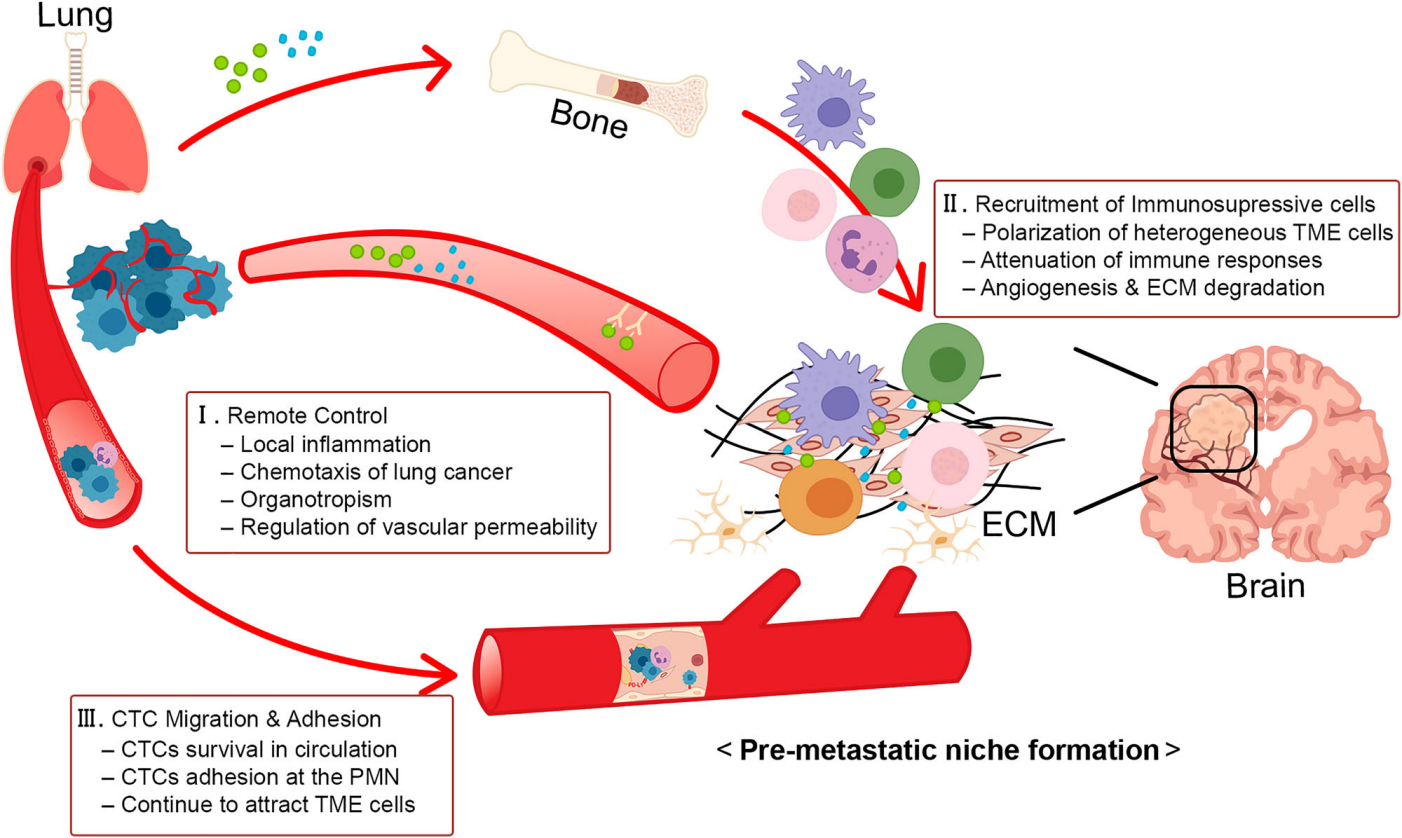
Process of PMN formation. PMN conditioning can be categorized into three sequential phases. (I) Remote control is the initial phase of the PMN induced by TDSFs and exosomes. (II) Recruitment of immunosuppressive cells is the second phase of PMN formation. TME cells exert both antitumor and protumor effects, with those displaying protumor activity classified as immunosuppressive cells. (III) CTC migration and adhesion is the final phase of the PMN. The establishment of the PMN serves as a preparatory process intricately linked to the subsequent steps of CTC survival, BBB penetration and their interactions with the brain microenvironment.

The PMN constitutes a noncancerous microenvironment formed in distant organs that facilitates the survival, growth and colonization of DTCs (Fig. 1). Remote control is the initial phase of the PMN induced by tumor-derived secreted-factors (TDSFs) and exosomes (Table 3). Recent reports indicate that lung cancer cells secrete inflammatory cytokines. Transforming growth factor-beta (TGFβ), a central mediator of EMT, induces local inflammation in distant target organs through the bloodstream and determines the specific sites for metastasis [14]. IL-6, a pro-inflammatory cytokine, plays a dual role, with sustained exposure potentially leading to protumor effects. In NSCLC, IL-6 induces TGFβ-mediated EMT through the IL-6/Janus kinase (JAK)/STAT signaling pathway, thereby enhancing metastatic potential via activation of nuclear factor kappa-B (NF-κB) signaling [15]. VEGF, an angiogenic factor, enhances ECM remodeling by stimulating the production of MMPs that are pivotal in the processes of cancer invasion and migration [16]. Collectively, these TDSFs are integral for the progression of NSCLC to BM.

**Table 3:** Function of TDSFs and exosomes in formation of the PMN.

	Molecule	Function	
TDSFs	TGFβ	Local inflammation, cancer invasion, cancer progression and TME cell differentiation	
	VEGF	Recruitment of myeloid cells and angiogenesis	
	IL-6	Cancer invasion, migration, angiogenesis and progression	
	IL-8	Angiogenesis	
	IL-10	Immunosuppression	
	IL-17	Indirect TME cell polarization	
	S100A8/A9	Immune cell chemotaxis	
	CXCR4	Cancer chemotaxis and extravasation	
	CCL2	Cancer invasion and recruitment of myeloid cells	
	**Molecule**	**Function**	**Mechanism**
Exosomes	miR-1260b	Cancer invasion	Upregulation of the WNT/β-catenin signaling pathway via targeting sFRP1 and Smad4
	miR-145		Upregulation of PRKCA
	MALAT1		Promoting the ERK/MAPK signaling pathway through upregulation of CXCL5 that targets the MALAT1 gene
	miR-21	TME polarization	Upregulation of the ERK/STAT3 pathway: TAM polarization
	LINC00482		Competitive binding to miR-142-30 and overexpression of TGFβ1: microglia polarization
	SOX2-OT		Inhibition of miR-627-3p activity that regulates the Smad pathway: TAM polarization
	miR-3157-3p	Regulation of vascular permeability	Targeting the TIMP/KLF2 axis for overexpression of VEGF, MMP-2, MMP-9 and TJ protein
	miR-23a		Targeting PHD1/2 and TJ protein ZO-1
	lnc-MMP-2		Suppression of miR-1207-5p activity and derepression of EPB41L5 that promotes EndMT

*TDSFs* tumor-derived secreted-factors, *TGF-β* transforming growth factor-beta, *EMT* epithelial mesenchymal transition, *VEGF* vascular endothelial growth factor, *PMN* pre-metastatic niche, *IL*-6 interleukin-6, *IL*-10 interleukin-10, *IL*-8 interleukin-8, *IL*-17 interleukin-17, *CXCR*4 C-X-C chemokine receptor type 4, *CCL2* C-C motif chemokine ligand 2, *sFRP1* secreted frizzled-related protein 1, *PRKCA* protein kinase C alpha, *MALAT*1 metastasis associated lung adenocarcinoma transcript 1, *ERK* extracellular signal-regulated kinase, *MAPK* mitogen-activated protein kinase, *CXCL5* C-X-C motif chemokine 5, *STAT3* signal transducer and activator of transcription, *TAM* tumor-associated macrophage, *TIMP*/*KLF2* tissue inhibitor of metalloproteinases/Krüppel-like factor 2, *MMP-2* matrix metalloproteinases-2, *MMP-9* matrix metalloproteinases-9, *TJ* tight junction, *PHD1*/*2* prolyl hydroxylase 1/2, *ZO*-1 zonula occludens-1, *EPB41L5* erythrocyte membrane protein band 4.1 like 5, *EndMT* endothelial-mesenchymal transition.

Exosomes are also critical in identifying and conditioning metastatic sites [17]. The specific integrins expressed on exosomal membranes largely determine the target organs. The metastasis-conducive microenvironment of these organs is shaped by Src phosphorylation and S100 gene expression, which are activated by exosomal integrins [18]. These processes contribute to the high tropism of lung cancer to metastasize to the brain. Exosomes carry a wide array of proteins, lipids and noncoding RNAs, which assist in traversing the BBB and augmenting the metastatic process. Their cargo includes miRNAs that regulate the invasive capacity and growth of NSCLC, as well as long noncoding RNAs (lncRNA) that modulate the polarization of TME cells [19–21]. During crossing of the BBB, specific miRNAs target angiogenesis-related factors, thereby regulating vascular permeability [22–24].

Recruitment of immunosuppressive cells is the second phase of PMN formation. TME cells exert both antitumor and protumor effects, with those displaying protumor activity classified as immunosuppressive cells. Lung cancer cells reprogram TME cells via TDSFs and exosomes, altering their phenotype and function to adopt an immunosuppressive state and evade immune surveillance [25]. These immunosuppressive cells facilitate metastasis by reducing immune activity in circulation and promoting the establishment of a supportive microenvironment in target organs [26–28].

CTC migration and adhesion is the final phase of the PMN. Immunosuppressive cells travel through the bloodstream in association with CTCs, leading to the establishment of CTM. These immune cells protect CTCs, promoting their survival in circulation. By evading immune detection with support from immunosuppressive cells, CTCs eventually reach the brain, where a favorable PMN has already been established [11].

### Conditioning of the immunosuppressive environment in the PMN

Myeloid-derived cells, including myeloid-derived suppressor cells (MDSCs), macrophages and neutrophils, exhibit functional diversity and phenotypic heterogeneity that facilitate spatial interactions between lung cancer and TME components, thereby augmenting BM [13].

MDSCs exhibit abnormal myeloid differentiation in the TME and play an important contributory role to the development of metastasis [29]. These cells are categorized into two primary groups: polymorphonuclear MDSCs (PMN-MDSCs) and monocytic MDSCs (M-MDSCs). M-MDSCs are capable of suppressing all types of T cell responses and produce nitric oxide (NO), along with various suppressive cytokines. On the other hand, PMN-MDSCs selectively inhibit antigen-specific T cell responses and display activity under restricted conditions [30]. MDSC-mediated immune suppression is facilitated by several key factors, such as TGFβ, IL-10, cyclooxygenase-2 (COX2) and arginase-1 (ARG-1). Recruitment and activation of MDSCs at PMN sites are driven by VEGF, S100A8/A9, IL-6, hypoxia-induced lysyl oxidase (LOX) and IL-10 [31]. Hypoxia-inducible factor (HIF)-1α serves as a crucial mediator that influences MDSC differentiation into tumor-associated macrophages (TAMs) by increasing the expression of inducible nitric oxide synthase (iNOS) and ARG, thereby augmenting their suppressive activity [32].

TAMs, a distinct subset of macrophages, are associated with tumor progression and play a crucial role in BM of NSCLC. TAMs exhibit heterogeneity, with certain subsets contributing to tumor progression and immune evasion, which can spatially induce hypoxia within TME [33]. TAMs are classified into two types: M1, which possesses antitumor functions, and M2, which is associated with protumor functions and activated in BM [34]. M1-type TAMs are involved in antigen presentation and immune surveillance, releasing pro-inflammatory cytokines (such as NO, tumor necrosis factor (TNF) and IL-6) in the early stages [34]. Conversely, M2-type TAMs impede the cytotoxic function of effector T cells by inhibiting antigen presentation while promoting regulatory T cell (T_reg_) differentiation through secretion of anti-inflammatory cytokines (such as IL-10 and TGFβ) [34, 35]. IL-10 secreted by M2 TAMs promotes NSCLC metastasis through the JAK/STAT, NF-κB and Notch signaling pathways [36]. NSCLC cells secrete IL-17 to recruit macrophages in the PMN, leading to the release of prostaglandin-E2 (PGE2) that drives their polarization toward the M2 phenotype [37]. In addition, NSCLC cells with EGFR mutations secrete exosomes containing SOX2-overlapping transcript (SOX2-OT) that regulate expression of Smads, in turn promoting M2 polarization [21].

Although neutrophils are recognized as immune cells, they can act as components of the TME by secreting inflammatory cytokines that support the survival of CTCs. Tumor-associated neutrophils (TANs) display both heterogeneity and plasticity and are classified into N1 TAN with antitumor activity and N2 TAN with protumor activity [38]. N2 TANs exhibit functional and morphological similarities to PMN-MDSCs and are implicated in cancer progression. TGFβ secreted by lung cancer induces neutrophil differentiation into the immunosuppressive N2 phenotype, characterized by elevated ARG production and attenuated effector T cell function [38]. The dual roles of myeloid cells highlight the complexity of their interactions within the TME. Owing to their heterotypic phenotypes and plasticity, targeting a single myeloid cell type is unlikely to effectively mitigate metastasis [26].

## Intravasation and migration of CTM in the circulation

### Intravasation and CTM formation

**Fig. 2:**
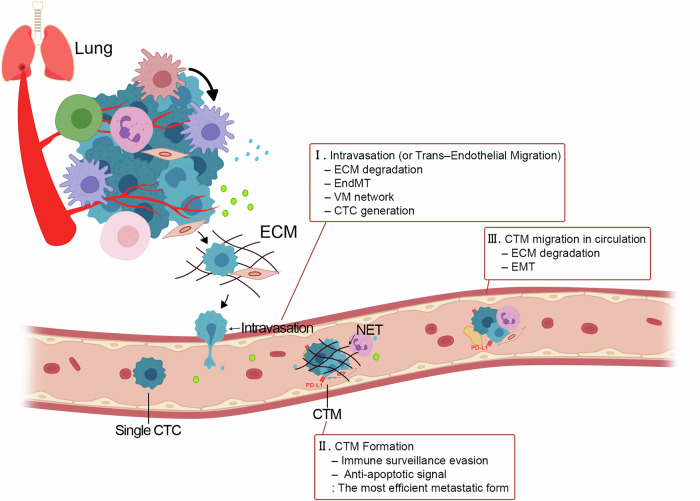
Intravasation and migration of CTM. Invasion and CTC survival can be categorized into three main steps. (I) Intravasation or trans-endothelial migration is the initial step of invasion. (II) CTM formation is crucial for CTC survival. CTM, which constitute aggregates of CTCs and TME cells, possess increased anti-apoptotic potential and superior anoikis resistance. (III) The migration of CTM is facilitated through active support provided by TME components within the cluster. CTM aggregates immune components, such as platelets and neutrophils, and stromal components, such as CAFs, which further enhance metastatic potential.

Intravasation or trans-endothelial migration is the initial step of invasion (Fig. 2). Lung cancer cells degrade the ECM and escape from the primary site to enter the bloodstream. This process is facilitated by interactions between cancer and endothelial cells, primarily through the cooperation of TGFβ-mediated Smad and Notch signaling pathways [39]. Endothelial cells undergo transition to acquire mesenchymal properties via the endothelial–mesenchymal transition (EndMT), similar to EMT of lung cancer during invasion. This transition is characterized by upregulation of vimentin and α-SMA, along with a decrease in TJ proteins, which are replaced by mesenchymal ECM proteins such as fibronectin, leading to increased vascular permeability [40].

Upon intravasation, CTCs form heterogeneous clusters of CTM to avoid immune surveillance until they reach the metastatic site. CTM, which constitute aggregates of CTCs and TME cells, possess increased anti-apoptotic potential and superior anoikis (detachment-induced cell death) resistance, thereby conferring a survival advantage [41]. The hypoxic environment within CTM augments metastatic potential, with HIF modulating tumor dissemination [10]. Within the CTM, heterogeneous expression of E-cadherin enables specific cells to retain cell–cell adhesion, preserving cluster integrity and facilitating the development of the most efficient metastatic phenotype [41].

CTM can migrate through ECM degradation and EMT. C-X-C chemokine receptor type 4 (CXCR4) secreted by cancer cells induces chemotaxis of CTM toward distant organs that serve as metastatic targets [42]. The CXCR4/C-X-C motif chemokine ligand 12 (CXCL12) signaling axis regulates lung cancer cell migration through the activation of Ras-related C3 botulinum toxin substrate 1 (RAC1), MMPs and multiple signaling pathways, including extracellular regulated protein kinase (ERK) and NF-κB [43]. Lung cancer cells undergoing EMT acquire enhanced migratory and invasive capabilities, along with stem cell-like properties, thereby increasing their metastatic potential. The migration of CTM is facilitated through active support provided by TME components within the cluster. CTM aggregates immune components, such as platelets and neutrophils, and stromal components, such as cancer-associated fibroblasts (CAFs), which further enhance metastatic potential (Table 4).

**Table 4:** Specific roles of cellular components in the formation of circulating tumor microemboli.

Cell	Cell markers	Secreted molecules	Cell-to-cell interaction	Roles in TME
Platelet	CD41^+^, CD61^+^	TGFβ, VEGF, PDGF MMP-9, IL-8, HGF	P-selectin (platelet)–PSGL-1 (cancer cell)	Immune surveillance evasion Physical stress resistance Invasion and migration Angiogenesis
N1 TAN	CD101^+^, CD10^+^, ICAM-1^+^	TNF, IL-1β ROS (high level-cytotoxic) S100A8/9		Immune surveillance Phagocytosis
N2 TAN	CD11b^+^, CD45^+^	ROS (low-level chronic) ARG, CCL2, CXCR4 VEGF, MMP-9	NET (endothelial cell–cancer cell)	Angiogenesis Chemotaxis Immune suppression Endothelial adherence
CAF	αSMA+	IL-6, TGFβ MMP-2, MMP-9 CXCL12	heterophilic N-cadherin/E-cadherin adhesion (CAF–cancer cell)	Invasion and migration ECM remodeling TME differentiation Angiogenesis

*TGF-β* transforming growth factor-beta, *VEGF* vascular endothelial growth factor, *PDGF* platelet-derived growth factor, *MMP-9* matrix metalloproteinases-9, *IL-8* interleukin-8, *HGF* hepatocyte growth factor, *PSGL* p-selectin glycoprotein ligand 1, *TAN* tumor-associated neutrophil, *ICAM-1* intercellular adhesion molecule 1, *TNF* tumor necrosis factor, *IL-1β* interleukin-1 beta, *ROS* reactive oxygen species, *ARG* arginase, *CCL2* C-C motif chemokine ligand 2, *CXCR4* C-X-C chemokine receptor type 4, *NET* neutrophil extracellular trap, *CAF* cancer-associated fibroblast, αSMA alpha smooth muscle actin, *IL-6* interleukin-6, *MMP-2* matrix metalloproteinases-2, *CXCL12*C-X-C motif chemokine ligand 12, *ECM* extracellular matrix, *TME* tumor microenvironment.

### Platelets: immune surveillance evasion in the CTM

Platelets protect lung cancer cells from shear stress conditions caused by blood flow, thereby inhibiting apoptotic signals. P-selectin expressed on activated platelets binds to p-selectin glycoprotein ligand 1 (PSGL-1) of NSCLC to form CTM, which serves as a physical shield [44]. ‘Platelet cloaking’ refers to the phenomenon in which platelets attach to antigen-presenting domains of CTCs, thereby allowing evasion of immune surveillance by effector T cells and natural killer (NK) cells [45]. This mechanism prevents cancer cell recognition and elimination through disrupting antigen presentation and lysis. Platelets serve as a reservoir for key immunosuppressive cytokines and upon activation, release α-granules containing TGFβ, VEGF and platelet-derived growth factor (PDGF) while simultaneously releasing dense granules to recruit further platelets [46]. Since CTCs may lose their invasive traits through mesenchymal–epithelial transition, platelets help to sustain EMT expression. Platelet-derived TGFβ and interactions between cancer cells and platelets concurrently activate Smad and NF-κB pathways, essential for maintaining the invasive properties of CTCs [47]. Platelet-derived extracellular vesicles enhance tumor proliferation via the MAPK signaling pathway and promote angiogenesis by stimulating angiogenic factors, such as MMP-9, IL-8 and hepatocyte growth factor (HGF). Expression of CD41, a platelet-derived integrin, on the surface of lung cancer cells enhances adhesion to endothelial cells and fibrinogen, further facilitating trans-endothelial migration [12].

### TANs: exploiting their intrinsic ability to support CTM

TANs assist cancer cells in infiltrating the bloodstream and facilitating the establishment of the most efficient CTM for metastasis [28]. HIF-1–VEGF signaling in CTM promotes neutrophil recruitment and adhesion at postcapillary venules [48]. Exosomal inflammatory chemoattractant protein S100A8/9, which is abundantly expressed at target sites of metastasis, promotes neutrophil chemotaxis [49]. Moreover, neutrophils have the ability to generate S100A8/9, activating the NF-κB, PI3K/AKT, mTOR and MAPK pathways that enhance cancer growth and invasion [50]. This mechanism leads to enhanced reactive oxygen species (ROS) production. Elevated ROS levels from N1 TANs are cytotoxic to tumor cells, whereas chronically low levels of ROS from N2 TANs induce genetic instability and immunosuppression, ultimately facilitating immune evasion of CTCs [51]. Neutrophils are polarized to the N2 TAN phenotype via Notch2–Jagged1 interaction of cancer cells and CAFs, accompanied by increased expression of ARG, C-C motif chemokine ligand 2 (CCL2), CXCR4 and MMP-9 [52]. N2 TANs support metastasis through generating neutrophil extracellular traps (NETs) to capture CTCs in circulation, enhancing their survival and facilitating endothelial attachment for extravasation [53].

### CAFs: structural support and enhancement of invasion of CTM

CAFs are activated fibroblasts that play a central role in shaping the TME. They interact with cancer cells directly via cell–cell adhesion and a range of secreted molecules, while also exerting indirect effects via ECM remodeling and immune cell infiltration. Exosomal miR-200 derived from cancer cells has the ability to convert normal fibroblasts into CAFs [54]. These CAFs contribute to CTM formation by connecting cancer cells via heterophilic N-cadherin/E-cadherin adhesion [55]. Integrins on CAFs interact with cancer cells and ECM components, enhancing the mobility of CTM. This cooperative interplay between CAFs and cancer cells is critical for the processes of migration and invasion. Exosomal miR-210 secreted by CAFs activates the PTEN/PI3K/AKT pathways, inducing EMT in NSCLC [56]. Autocrine secretion of IL-6 enhances the communication between lung cancer cells and fibroblasts by forming a positive feedback loop with TGFβ, thereby promoting EMT [57]. CAFs also secrete MMP-2 and MMP-9 that degrade the ECM to create gaps in the basement membrane and align fibronectin to support cancer cell invasion [55]. Furthermore, CAFs drive vascular mimicry (VM) network, forming capillary-like structures with loose junctions to facilitate intravasation and extravasation of cancer cells and TANs [52].

## BBB penetration

### The process of BBB penetration

Upon reaching the brain via the bloodstream, cancer cells encounter the BBB. Given that inflammation and immune responses are detrimental to neuronal survival, the BBB serves as a protective barrier limiting immune cell infiltration into the brain, but does not create a completely immune-privileged site [58].

**Fig. 3:**
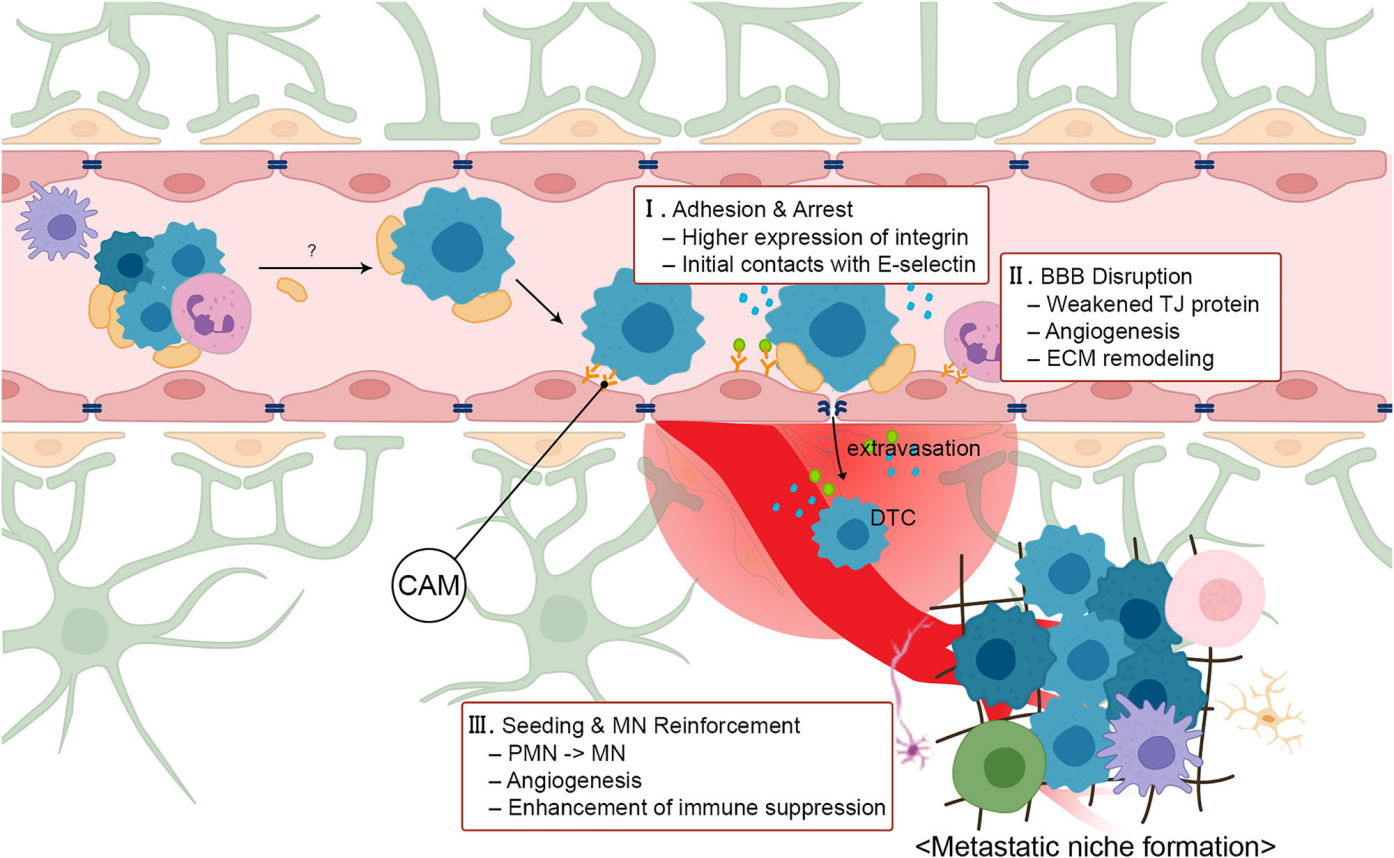
The process of BBB penetration. (I) CTCs adhere to endothelial cells by expressing adhesion molecules that facilitate extravasation. (II) The integrity of the BBB is disrupted by inflammatory and angiogenic factors. (III) Seeding and MN reinforcement occur after extravasation DTCs are dispersed within the PMN, which subsequently evolves into a MN. Reciprocal interactions among TME components further reinforce the MN and facilitate the outgrowth of lung cancer cells in the secondary organ.

The mechanisms by which cancer cells penetrate the BBB and invade the brain exploit the homing of immune cells to inflammatory (Fig. 3). CTCs adhere to endothelial cells by expressing adhesion molecules that facilitate extravasation. Notably, activated leukocyte cell adhesion molecule (ALCAM) has been identified as a key adhesion molecule mediating trans-endothelial migration and vascular co-option in NSCLC. ALCAM enhances the interaction between NSCLC cells and endothelial cells, ultimately augmenting the likelihood of BM formation [59]. Furthermore, NSCLC-derived factors, such as VEGF, insulin-like growth factor-binding protein 7 (IGFBP7), cystatin L, cathepsin C and TNF, degrade the glycocalyx, exposing E-selectin on the endothelial cell surface [60]. Increased exposure of E-selectin enhances the initial contacts between endothelial cells and lung cancer by mediating adhesion of CD15, which is overexpressed on the surface of metastatic cells [61].

The integrity of the BBB is disrupted by inflammatory and angiogenic factors. Lung cancer-derived TGFβ signaling modifies TJ proteins during localized inflammation at the BBB, increasing vascular permeability. TGFβ1-mediated exosomal lnc-MMP-2 suppresses miR-1207-5p activity, leading to disruption of TJs and a decline in barrier integrity [24]. In addition, NSCLC promotes the expression of AKR1B10, which activates the MAPK pathway, leading to MMP-2 and MMP-9 upregulation and further TJ disruption [62]. Tumor-induced angiogenesis is another major contributor to increased vascular permeability through disruption of endothelial cell junctions and promotion of both intravasation and extravasation. NSCLC-mediated PI3K/AKT, RAS/ERK and STAT3 signaling pathways are correlated with VEGFA upregulation. Upregulation of VEGFA stimulates the secretion of angiogenic factors, such as HGF, PDGF-BB, angiopoietin 2 (ANGPT2), follistatin, granulocyte colony-stimulating factor (G-CSF) and IL-8 [12]. Disintegrin and metalloprotease 9 (ADAM9) plays a role in regulating the expression of VEGFA and ANGPT2 in endothelial cells [63]. These molecular events collectively contribute to vascular leakiness, enabling cancer cells to breach the BBB.

Seeding and metastatic niche (MN) reinforcement occur after extravasation. Once extravasation has occurred, DTCs are dispersed within the PMN, which subsequently evolves into a MN. Reciprocal interactions among TME components further reinforce the MN and facilitate the outgrowth of lung cancer cells in the secondary organ. Cells within the TME that contribute to BBB penetration include BBB-resident cells, such as endothelial cells, pericytes, and astrocytes, as well as TAMs, which play a pivotal role in inducing BBB hyperpermeability (Table 5). Having addressed the role of endothelial cells in CTC adhesion and extravasation above, the following section will focus on the contribution of other TME components.

**Table 5:** Cell-mediated modulation of BBB integrity.

Cell	Cell markers	Secreted molecules	Cell-to-cell interaction	Roles in TME
Endothelial cell	ALCAM^+^ E-selectin^+^	VEGFA ANGPT2 PDGF-BB	ALCAM (cancer cell)–ALCAM (endothelial cell) CD15 (cancer cell)–E-selectin (endothelial cell)	Cancer cell adhesion Angiogenesis Extravasation Invasion and migration
Pericyte	PDGFRβ^+^ Desmin^+^	ECM protein (collagen, fibronectin) MMP-9	N-cadherin (pericyte)–N-cadherin (cancer cell) Decreased N-cadherin (pericyte)–N-cadherin (endothelial cell)	Vascular structure remodeling Angiogenesis Invasion and migration
Astrocyte	GFAP^+^	TNF, IL-6 MMP-2, MMP-9 CCL2	–	ECM remodeling Tumor growth and proliferation Recruitment of immune cells
M2 TAM	CD163^+^, CD206^+^,	ARG-1, ornithine, polyamine MMP-9 VEGF, PDGF, IGF, CXCL12	CCR2(TAM)–CCL2(astrocyte)	ECM remodeling Angiogenesis Recruitment of immune cells TME differentiation

### Pericytes and astrocytes: failure to maintain BBB integrity

Pericytes play a crucial role in restricting cancer cell metastasis. In NSCLC, there is a notable reduction in the number of platelet-derived growth factor receptor beta (PDGFRβ)-positive pericytes, which are essential for vascular transport regulation, resulting in increased BBB permeability [64]. Lung cancer-derived TGFβ activates the Smad2/3 and Akt/mTOR pathways in pericytes, inducing their transition into myofibroblasts that excessively produce ECM proteins such as collagen and fibronectin, in turn ultimately causing structural alterations in blood vessels and cancer cell invasion [65]. Overexpression of desmin-positive pericytes leads to acquisition of a more myofibroblast-like phenotype [8]. The loss of pericytes weakens endothelial cell TJs, leading to endothelial hyperplasia and abnormal angiogenesis. Reduced levels of N-cadherin, an adhesion molecule of pericytes and endothelial cells, coupled with aberrant expression on metastatic cancer cells facilitate the process of trans-endothelial migration [66].

NSCLC establishes BMs through bidirectional interactions with activated astrocytes. Initially, NSCLC cells release IL-8, macrophage migration inhibitory factor (MIF) and plasminogen activator inhibitor-1 (PAI-1), which activate astrocytes. In response, activated astrocytes produce inflammatory cytokines, such as TNF and IL-6, which stimulate cancer cells and promote MMP-2 and MMP-9 secretion via the action of urokinase-type plasminogen activator (uPA) [67]. In addition, astrocyte elevated gene 1 (AEG-1) is upregulated in NSCLC cells, which is associated with increased MMP-9 secretion in the TME [68]. Persistent MMP secretion driven by uPA and AEG-1 further promotes BBB degradation, supporting subsequent tumor growth and proliferation via angiogenesis. Furthermore, astrocytic sphingosine 1-phosphate 3 (S1P3) mediates IL-6 and CCL2 expression, promoting the recruitment of myeloid cells, consequently weakening endothelial cell adhesion and enhancing permeability [69].

### TAMs: facilitators of extravasation

TAMs promote cancer invasiveness and metastatic potential through secretion of various factors that activate signaling pathways involved in ECM degradation and angiogenesis [12]. M2-type TAMs enhance ARG-1 activity, inducing the biosynthesis of ornithine and polyamines, which contribute to ECM remodeling [70]. In addition, M2 TAMs produce MMP-9, which disrupts the ECM and TJs, thus creating intercellular gaps [71]. Tissue damage triggers the release of inflammatory cytokines and chemokines and inflammation-related pathways such as NF-κB and Wnt signaling [72]. These responses facilitate immune cell recruitment, and the amplified immune activity of recruited cells further exacerbates BBB hyperpermeability. M2 TAMs express high level of VEGF, PDGF and insulin-like growth factor (IGF) that collectively stimulate angiogenic processes [35]. VEGF and CXCL12 recruit and differentiate myeloid cells, disrupting the integrity of BBB vessels during angiogenesis and contributing to increased vascular permeability [73].

## Colonization in the brain

### The stage of completion of metastasis

**Fig. 4:**
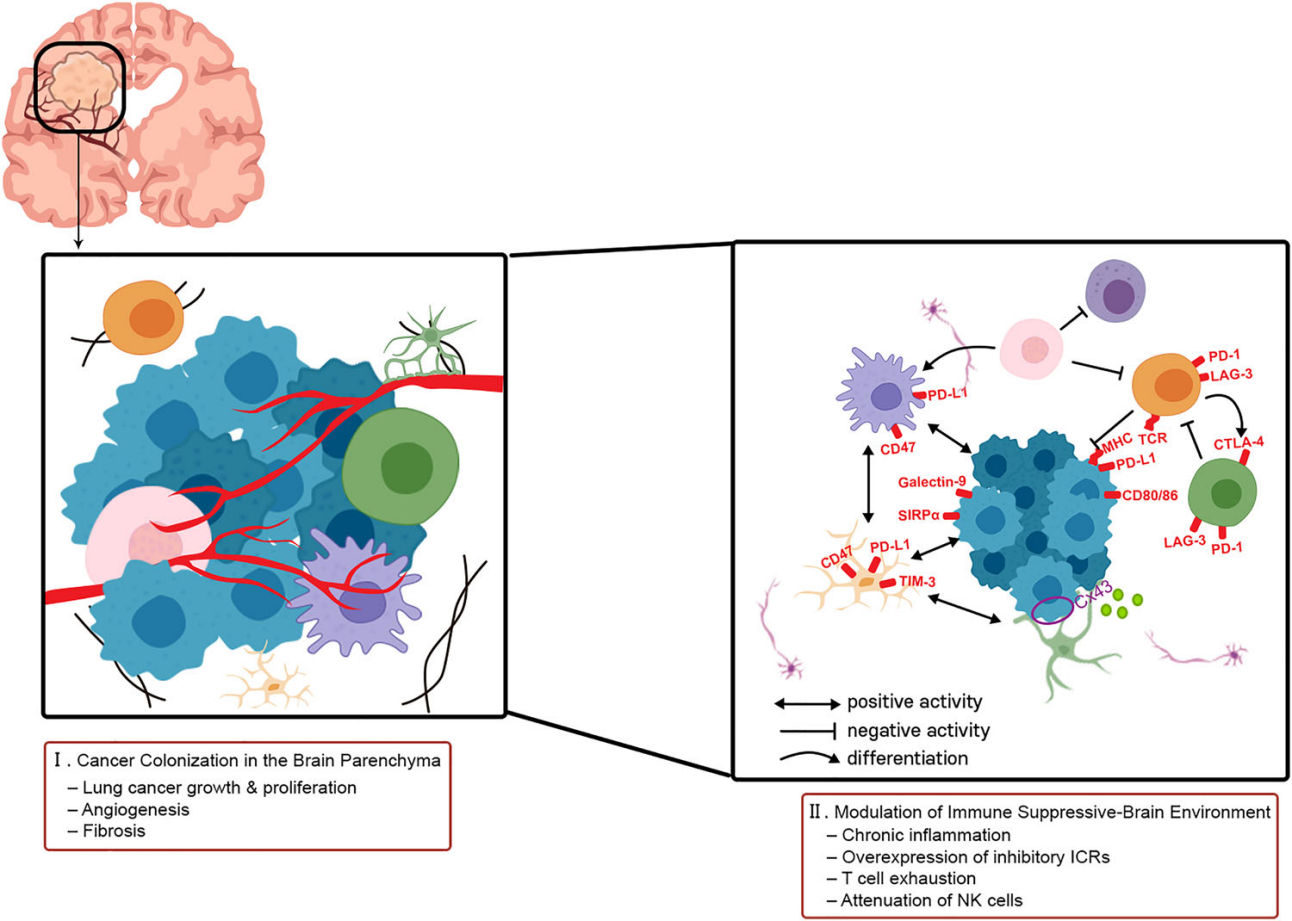
Two key interactions with the brain microenvironment during colonization. (I) Cancer colonization is influenced by tumor-promoting signals and supporting mechanisms in the brain. Angiogenesis is essential to supply oxygen and nutrients necessary for proliferation of metastatic lung cancer cells. To sustain robust proliferation, lung cancer cells augment stiffness of the surrounding matrix in the MN. (II) Interactions between metastatic lung cancer cells and brain TME are essential for successful colonization. This process involves not only resident brain cells (such as astrocytes and microglial cells) but also immune cells (such as macrophages, effector T cells and T_regs_) that originate in bone and migrate to the brain through the bloodstream. MHC major histocompatibility complex class, TCR T cell receptor.

Lung cancer cells exhibit phenotypic plasticity that enable their adaptation to the metastatic brain environment through the acquisition of neural- or stem cell-like features. This phenotypic switch not only enhances tumorigenicity but also facilitates immune evasion in the brain. Effective colonization and sustained proliferation in the MN depend on reciprocal interactions with the brain microenvironment (Fig. 4).

Cancer colonization is influenced by tumor-promoting signals and supporting mechanisms in the brain. In NSCLC, overexpression of brain-derived neurotrophic factor (BDNF), a neurotrophin implicated in brain pathology, enhances metastatic cell settlement. BDNF promotes STAT3 activity, stimulating the PI3K/AKT and MAPK pathways, both of which function in anti-apoptotic signaling in lung cancer and facilitate successful colonization in the MN [74]. In addition, angiogenesis is essential to supply oxygen and nutrients necessary for proliferation of metastatic lung cancer cells. NSCLC promotes the production of angiogenic mediators through multiple interplays with TME cells. To sustain robust proliferation, lung cancer cells augment stiffness of the surrounding matrix in the MN, thereby limiting T cell infiltration [75]. Through interactions with cancer cells, reactive astrocytes, vesicular pericytes and fibroblasts overexpress fibrosis-related genes [13, 75]. Lung cancer growth at the secondary site is effectively sustained via comprehensive conditioning.

The crosstalk between cancer cells and TME cells plays a crucial role in modulating the immunosuppressive milieu of the brain. Chronic exposure to tumor antigens and inflammatory cytokines drives abnormal immune cell activation. Inhibitory immune checkpoint receptors (ICRs), such as programmed cell death protein 1 (PD-1) and lymphocyte activation gene-3 (LAG-3), are primarily expressed on CD8⁺ cytotoxic T cells and T_regs_. In addition, T cell immunoglobulin and mucin-domain containing-3 (TIM-3) is predominantly expressed in innate immune cells, including NK cells, macrophages and dendritic cells [76]. They can regulate immune responses for conditioning immune suppression. MDSCs secrete immunosuppressive cytokines, promote T_reg_ differentiation and maintain consistently high expression of PD-L1, thereby weakening NK cell cytotoxicity [26]. This process involves not only resident brain cells (such as astrocytes and microglial cells) but also immune cells (such as macrophages, effector T cells and T_regs_) that originate in bone and migrate to the brain through the bloodstream (Table 6).

**Table 6:** Cellular contributors to brain colonization.

Cell	Cell markers	Secreted molecules	Cell-to-cell interaction	Roles in TME
Astrocyte	GFAP^+^, S100β^+^	PA		Tumor cell apoptosis Suppression of cancer progression and metastasis
	GFAP^+^, CD274^+^	ET-1 TNF, IL-6 CCL2	Cx43 gap junction (astrocyte–cancer cell)	Tumor growth Chemotaxis Immune suppression
TAM-BMDM	Iba1^+^, CD49d^+^, Tmem119^−^, P2ry12^−^, Siglech^−^, Sall1^−^	Midkine IL-10, TGFβ	PD-1 (CTL)–PD-L1 (TAM) TIM-3 (TAM)–Galectin-9 (cancer cell) SIRPα (TAM)–CD47 (cancer cell)	Immune suppression Tumor survival TME differentiation
TAM-MG	Iba1^+^, CD49d^−^, Tmem119^+^, P2ry12^+^, Siglech^+^, Sall1^+^	IL-6, IL-8, IGF, CCL20	PD-1 (CTL)–PD-L1 (TAM) TIM-3 (TAM)–Galectin-9 (cancer cell) SIRPα (TAM)–CD47 (cancer cell)	Invasion and migration Tumor survival Immune suppression
T cell	CD3^+^, CD4^+^, CD8^+^	IFNγ	PD-1 (T cell)–PD-L1 (cancer cell) PD-1 (T cell)–PD-L1 (TAM) LAG-3 (T cell)–MHC-II (cancer cell) TCR (T cell)–MHC (cancer cell)	Immune response
T_reg_	CD4^+^, CD25^+^, FOXP3^+^	IL-10, TGFβ	PD-1 (T_reg_)–PD-L1 (cancer cell) LAG-3 (T_reg_)–MHC-II (cancer cell) CTLA-4 (T_reg_)–CD80/86 (cancer cell)	Immune suppression TME differentiation

*Iba1* ionized calcium-binding adaptor molecule 1, *PA* plasminogen activator, *P2ry12* purinergic receptor *P2Y12*, *Sall1*spalt-like transcription factor 1, *siglech* sialic acid-binding Ig-like lectin H, *Tmem119* transmembrane protein 119.

### Astrocytes: a key component for BM at the colonization stage

Astrocytes initially act to suppress tumor growth during the early stages of BM. Glial fibrillary acidic protein (GFAP)⁺/S100β⁺ astrocytes secrete PA, which induces tumor cell apoptosis and limits the activation of L1CAM to restrict tumor dissemination. However, lung cancer cells counteract this by producing anti-PA serpins, which enable apoptotic signal evasion and vascular co-option [77]. In addition, astrocytes release exosomes containing miR-142-3p, which downregulates the expression of transient receptor potential ankyrin-1 (TRPA1) in lung cancer cells, thereby disrupting the TRPA1–FGFR2 axis and inhibiting tumor progression and metastasis [78].

Astrocytes that exert antitumor effects can undergo polarization to a protumor state, transforming into cancer-associated GFAP⁺/CD274⁺ astrocytes through damage-associated signals and interactions with microglia [79]. Lung cancer cells stimulate the expression of endothelin-1 (ET-1) in astrocytes, activating PI3K/AKT and MAPK pathways [80]. Furthermore, lung cancer cells communicate with reactive astrocytes to upregulate protocadherin 7 (PCDH7), which promotes the formation of connexin 43 (Cx43) gap junctions, allowing the transfer of cyclic GMP–AMP (cGAMP) into astrocytes. The presence of cGAMP triggers the STING/STAT3 pathway that serves as a key protumor signaling within metastatic lesions [81]. Furthermore, astrocytes transfer PTEN-targeting miR-19a via exosomes, promoting PTEN loss in cancer cells, in turn enhancing CCL2 secretion and promoting chemotaxis of ionized calcium-binding adapter molecule 1 (IBA1)⁺ myeloid cells [82].

### TAMs of diverse origins: immunosuppressive effects in the brain microenvironment

TAMs exert strong immunosuppressive effects in patients with BM. These cells are characterized by high phenotypic plasticity and contribute to shaping immunosuppressive TME. Within the brain, TAMs are primarily categorized into two major subsets: bone marrow-derived (TAM-BMDMs) and microglia-derived (TAM-MGs). TAM-MGs are enriched in genes related to pro-inflammatory cytokines and chemokines, whereas TAM-BMDMs are characterized by their anti-inflammatory and immunosuppressive properties [83]. TAM-MGs typically reside at the lesion periphery, whereas TAM-BMDMs are predominantly located within the tumor core, acting as a central component of the TME [84].

TAM-BMDMs fulfill the roles of conventional TAMs described earlier. TAM-BMDMs infiltrate the metastatic niche by traversing the BBB and establish a positive feedback loop with the brain microenvironment, thus enhancing immunosuppression. For instance, M2-like TAMs promote T_reg_ differentiation that, in turn, supports further M2 polarization [35]. CD74⁺ TAMs bind to MIF secreted by pSTAT3⁺ astrocytes, leading to the induction of midkine, a key mediator of NSCLC proliferation [85]. Moreover, TAMs suppress T cell expansion via persistent expression of inhibitory receptors and production of suppressive mediators, such as IL-10 and TGFβ, which contribute to T cell exhaustion [86].

Similar to their BMDM counterparts, TAM-MGs play dual roles. M1-like microglia secrete NO to lyse tumor cells and promote cytotoxic T cell activation through antigen presentation. Conversely, M2-like microglia secrete IL-6, IL-8, IGF and CCL20, which aid in maintaining lung cancer stemness and immunosuppressive cell recruitment [87]. NSCLC-derived soluble factors, such as LINC00482, can promote M2 polarization of microglial cells [20]. Interactions between CD47 expressed by tumor cells and microglial signal regulatory protein α (SIRPα) suppress phagocytosis, facilitating metastatic colonization [88].

### T cells: a main regulator of immune responses in immune-privileged site

NSCLC cells suppress antigen presentation, restrict lymphocyte infiltration into the brain and impair T cell function to maintain an immunosuppressive state. Endothelial cells modulate immune cell infiltration by downregulating vascular cell adhesion molecule 1 (VCAM-1), leading to reduced lymphocyte adhesion and subsequent trans-endothelial migration [89]. While ICRs such as PD-1, LAG-3 and TIM-3 have traditionally been considered markers of T cell exhaustion, recent evidence indicates that their expression levels could also reflect an autoregulatory mechanism in activated tumor-infiltrating lymphocytes (TILs). Therefore, in the immune-privileged environment of the brain, high ICR expression does not necessarily indicate immune dysfunction but may potentially reflect active T cell immune responses [90]. Notably, EGFR- and KRAS-mutant NSCLC cases typically exhibit a reduced proportion of TILs and corresponding low ICR expression, indicating a deficiency in immune engagement rather than a fully functional T cell population [76]. Moreover, chronic inflammation coupled with sustained exposure to immunosuppressive cytokines further impairs T cell function, leading to reduced cytotoxic activity and diminished IFNγ production [86].

TME-derived TGFβ signaling promotes the differentiation of T cells into T_reg_ cells, leading to the acquisition of immunosuppressive activity [35]. T_regs_ are recruited to PMNs, thereby establishing an environment conducive to BM. Forkhead box P3 (FOXP3)⁺ T_regs_ are characterized by elevated expression of cytotoxic T-lymphocyte-associated protein 4 (CTLA-4), PD-1 and TNF receptors, which collectively function to limit immune responses [91]. The primary immunosuppressive mediators secreted by T_regs_, IL-10 and TGFβ attenuate effector T cell activity and modulate TME polarization, playing a central role in maintaining the immunosuppressive microenvironment of the brain [92]. These cells actively infiltrate the brain and inhibit antitumor immune responses, suggesting that targeting T_regs_ could serve as a promising strategy to prevent or limit BM.

## Clinical implication and future perspective

### ICIs in NSCLC BMs

Immune checkpoint inhibitors (ICIs) have emerged as a promising systemic strategy for the management of BM in NSCLC. Clinical trials, including CheckMate 227, KEYNOTE-189 and KEYNOTE-024, as well as the ATEZO-BRAIN study, enrolled patients with treated and stable BM and demonstrated comparable intracranial and extracranial benefits with ICIs such as nivolumab, pembrolizumab and atezolizumab [93–95]. ICIs can induce clinically meaningful intracranial responses, particularly in patients with PD-L1-positive tumors, although responses may occur more slowly compared to targeted therapies. Combining stereotactic radiosurgery with ICIs may enhance intracranial disease control, although optimal sequencing strategies remain to be established [93–95]. Collectively, these findings underscore that ICIs can achieve durable and clinically relevant outcomes in patients with NSCLC and BM. Nevertheless, the intracranial immune microenvironment presents unique barriers: it is characterized by the restrictive BBB, a paucity of TILs and an increased presence of immunosuppressive cells, including microglia and astrocytes. While focal BBB disruption by tumor invasion may partially permit drug entry, both T cell trafficking and ICI penetration remain limited. Thus, the immune microenvironment continues to pose substantial therapeutic challenges, highlighting the need for further research focused on the TME in NSCLC BM.

### Drug delivery strategies across the BBB

Drug delivery across the BBB in lung cancer BM remains a critical therapeutic challenge and several strategies are under investigation to overcome this obstacle. One approach is the use of BBB-penetrant small-molecule tyrosine kinase inhibitors, such as osimertinib, lorlatinib and alectinib, which are specifically designed to achieve central nervous system (CNS) penetration [96]. Biological pathways can also be exploited, including receptor-mediated transcytosis, where therapeutic agents utilize transporters such as the transferrin receptor, or engineered ‘shuttles’ such as Angiopep-2 conjugates and transferrin receptor bispecifics. Carrier-mediated transport pathways, such as uptake via the large neutral amino acid transporter 1 (LAT1), represent another promising mechanism for enhancing CNS drug access [96]. Physical strategies, including focused ultrasound in combination with microbubbles or electrical field-based disruption, have been shown to transiently and reversibly open the BBB, thereby facilitating drug delivery. In addition, nanotechnology-based carriers—such as nanoparticles, liposomes and exosomes—are being investigated to transport therapeutic agents across the BBB in a more targeted fashion [97]. Alternative routes of administration, such as intrathecal or intraventricular injection into the cerebrospinal fluid or intranasal delivery that bypasses systemic circulation, are being explored as complementary approaches to directly reach the brain parenchyma. These strategies highlight the diversity of ongoing efforts to overcome BBB-related barriers and improve the efficacy of systemic therapy in lung cancer BM.

### Potential blood biomarkers for BM in NSCLC

Recent advances highlight the promise of blood-based biomarkers for the detection and monitoring of BM in NSCLC based on the analysis of CTCs and tumor-derived molecules such as circulating tumor DNA, circulating tumor RNA, proteins and microvesicles including exosomes [98–100]. Genetic biomarkers encompass a broad spectrum of DNA alterations, ranging from point mutations to chromosomal structural changes [98]. Beyond genomics, epigenomic profiling of plasma cell-free DNA methylation patterns have emerged as a powerful tool, capable of distinguishing BM from primary CNS tumors. Protein-based biomarkers are also gaining traction: recent studies demonstrated that serum neurofilament light chain (sNfL) and GFAP correlate with the presence and progression of BM, while proteomic analyses identified serum cathepsin F and fibulin-1 as diagnostic and response markers, and exosomal proteins such as MUC5B and SELL as BM-associated signatures [99]. In parallel, ncRNAs, including miRNAs and lncRNAs, have been implicated as key regulators of tumor–microenvironment interactions and potential minimally invasive biomarkers for BM [100]. Collectively, these findings underscore that blood-based biomarkers offer a noninvasive means for early detection, real-time monitoring and therapeutic stratification in NSCLC BM, while emphasizing the need for prospective validation in clinical trials.

## Conclusions

This review focuses specifically on NSCLC, delving into the mechanisms underlying BM that represents one of the most clinically challenging aspects of lung cancer treatment. In particular, we explore how the TME facilitates and regulates BM, with specific focus on the strategies employed by NSCLC cells to actively exploit this environment. Although the significance of the TME is well recognized, our current knowledge remains fragmented, with the majority of existing studies concentrating on isolated components rather than offering a comprehensive and integrative overview. Given that the metastatic behavior of cancer cells within the brain cannot be fully explained by tumor-intrinsic factors alone, future research should prioritize elucidation of the complex crosstalk between tumor cells and TME to facilitate the design of more effective targeted therapeutic strategies for BM.

## Supplementary information

Supplementary Information
