# Supplementary material for: Brain metastasis from non-small cell lung cancer: crosstalk between cancer cells and tumor microenvironment components
Source: Exp Mol Med. 2025 Dec 22;57(12):2749–62. doi: 10.1038/s12276-025-01604-z (PMC12800198; doi:10.1038/s12276-025-01604-z)

Supplementary information

Supplementary Figure 1. The cells and components involved in the process of brain metastasis from non-small cell lung cancer

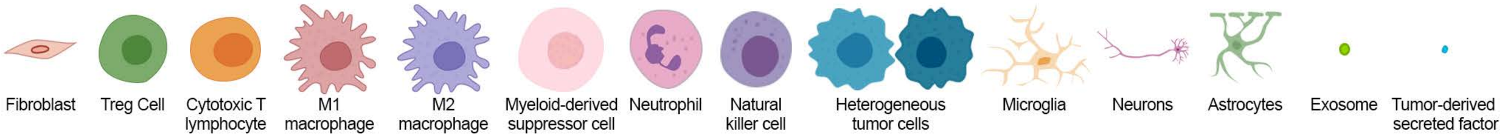

Supplement: Supplementary file 1 — Supplementary Information [file 12276_2025_1604_MOESM1_ESM.pdf]
